# Hypocholesterolemic agents and the gut microbiota: a review of interactions and modulatory activity

**DOI:** 10.1007/s43440-026-00874-2

**Published:** 2026-06-15

**Authors:** Manuel Mântua E. Garcia, Virginie Xavier, Ricardo C. Calhelha, Sandrina A. Heleno

**Affiliations:** CIMO, LA SusTEC, Instituto Politécnico de Bragança, Campus de Santa Apolónia, Bragança, 5300- 253 Portugal

**Keywords:** Gut microbiota, Cholesterol, Modulatory activity, Hypocholesterolemic agents, Bioavailability

## Abstract

Hypercholesterolemia is a major risk factor that contributes to the development and/or aggravation of cardiovascular diseases. High cholesterol levels are frequently managed with hypocholesterolemic agents, either synthetic or natural. Beyond their cholesterol-lowering effects, these compounds can also affect the host’s gut microbiota. This review examined the extent and quality of the modulatory activity of selected hypocholesterolemic agents (*n* = 11) on the gut microbiota profile, based on a comprehensive literature search and analysis of interactions between the gut microbiota and distinct cholesterol-lowering compounds. With a few exceptions, anti-cholesterol interventions were associated with changes in gut microbiota β-diversity and a decrease in the *Bacillota*/*Bacteroidota* ratio. Further analyses at family and genus levels revealed patterns of modulation that clustered by molecular target and bioavailability. Overall, gut microbiota shifts favoured short-chain fatty acid (SCFA)-producing bacteria over inflammation-promoting taxa. Furthermore, for some anti-cholesterol compounds, microbial metabolism and the concomitant release of bioactive metabolites suggested partially microbiota-dependent cholesterol-lowering effects. These findings may inform new strategies for cholesterol management from a gut-health perspective; however, further research is needed to establish causality and draw robust conclusions regarding medication–microbiota relationships.

## Clinical trial number

Not applicable.

## Introduction

Cholesterol is an essential lipophilic molecule, integral in providing rigidity, fluidity, and permeability to the cellular membranes of animals. It is also a precursor of steroid hormones, bile acids, and fat-soluble vitamins, also contributing to maintaining physiological brain functions [1]. Because of these roles, cholesterol homeostasis is critical, both excess and deficiency are linked to disease [2]. Elevated cholesterol is associated with increased cardiovascular mortality and is used as a primary lipid biomarker for cardiovascular risk assessment [3]. Accordingly, hypocholesterolemic agents are widely used to treat hypercholesterolemia and restore overall lipid balance.

Current cholesterol-lowering therapies include several classes of synthetic drugs and natural agents with distinct efficacy profiles and molecular targets. Among synthetic therapies, statins inhibit activity of hydroxyl-methyl-glutaryl-coenzyme A reductase (HMGCR), reducing hepatic cholesterol synthesis [4]; ezetimibe blocks sterol transporter Niemann-Pick C1-like 1 (NPC1L1), decreasing cholesterol absorption [5]; bile acid sequestrants (BAS) bind intestinal bile acids (BAs), increasing fecal BA excretion and upregulating cholesterol 7 alpha-hydroxylase (CYP7A1) that converts cholesterol into BAs [6]; and fibrates act as peroxisome proliferator-activated receptor alpha (PPAR-α) agonists, enhancing β-oxidation of fatty acids, mainly lowering triglyceride (TG) and very low-density lipoprotein (VLDL) synthesis [7]. Similar mechanisms are reported for natural agents: phytosterols (PS) competitively inhibit NPC1L1-mediated cholesterol transport [8]; red yeast rice (RYR) contains a lovastatin-like compound and thus acts primarily as an HMGCR inhibitor [9], epigallocatechin gallate (EGCG) inhibits HMGCR via cofactor-competitive binding [10]; and phenolics such as curcumin and quercetin may act as partial PPAR-α agonists, mimicking fibrate-like effects [11, 12]. Finally, β-glucans increase BA excretion by forming glucan-biliary salt complexes [13] and may also indirectly support cholesterol management via prebiotic effect on gut microbiota.

The human gut microbiota comprises all microorganisms that colonize the gastrointestinal (GI) tract, including, but not limited to, bacteria, yeasts, and viruses. Concentrated mainly in the colon, these GI tract communities interact symbiotically with the host, and support key functions such as gut barrier integrity [14], protection against pathogens [15], and cholesterol homeostasis [16]. In particular, commensal bacteria can regulate cholesterol through metabolites that influence cholesterol-related enzymes and gene expression, as detailed in section “Overview of gut microbiota-cholesterol interactions”. Furthermore, growing evidence links gut microbiota composition to disease [17] and variability in medication responses [18].

Medication-microbiota interactions are complex and bidirectional. The gut microbiota profile, i.e., the symbiotic community of microbial species, can be affected by medication intake, shifting composition by inhibiting or promoting specific taxa [19]. In turn, the microbiome can metabolize drugs, influencing individual responses and efficacy [20]. Despite this, the effects of cholesterol-lowering therapies on gut microbial population and diversity have yet to be fully explored. This review focuses on the modulatory effects of hypocholesterolemic agents on the gut microbiota and their downstream effects, including changes in metabolite, enzyme and gene expression. Agents were selected based on: (i) clinically relevant cholesterol-lowering effects supported by randomized controlled trials and systematic reviews; and (ii) evidence of gut microbiota modulation, tested in vitro and/or in vivo. Obeying these criteria, we selected 11 agents: ezetimibe, bile acid sequestrants, fenofibrate, phytosterols, soy isoflavones, red yeast rice, β-glucans, curcumin, quercetin, EGCG, and berberine. Considering the availability of exploratory and review studies focused on statins’ impact on gut microbiota [21–24], this cholesterol-lowering drug was excluded. This review first contextualizes cholesterol management in dysbiotic and eubiotic settings, then summarizes microbiota modulation by cholesterol-lowering agents, grouped by primary molecular target, and finally discusses emerging metabolite-related associations.

## Overview of gut microbiota-cholesterol interactions

**Fig. 1:**
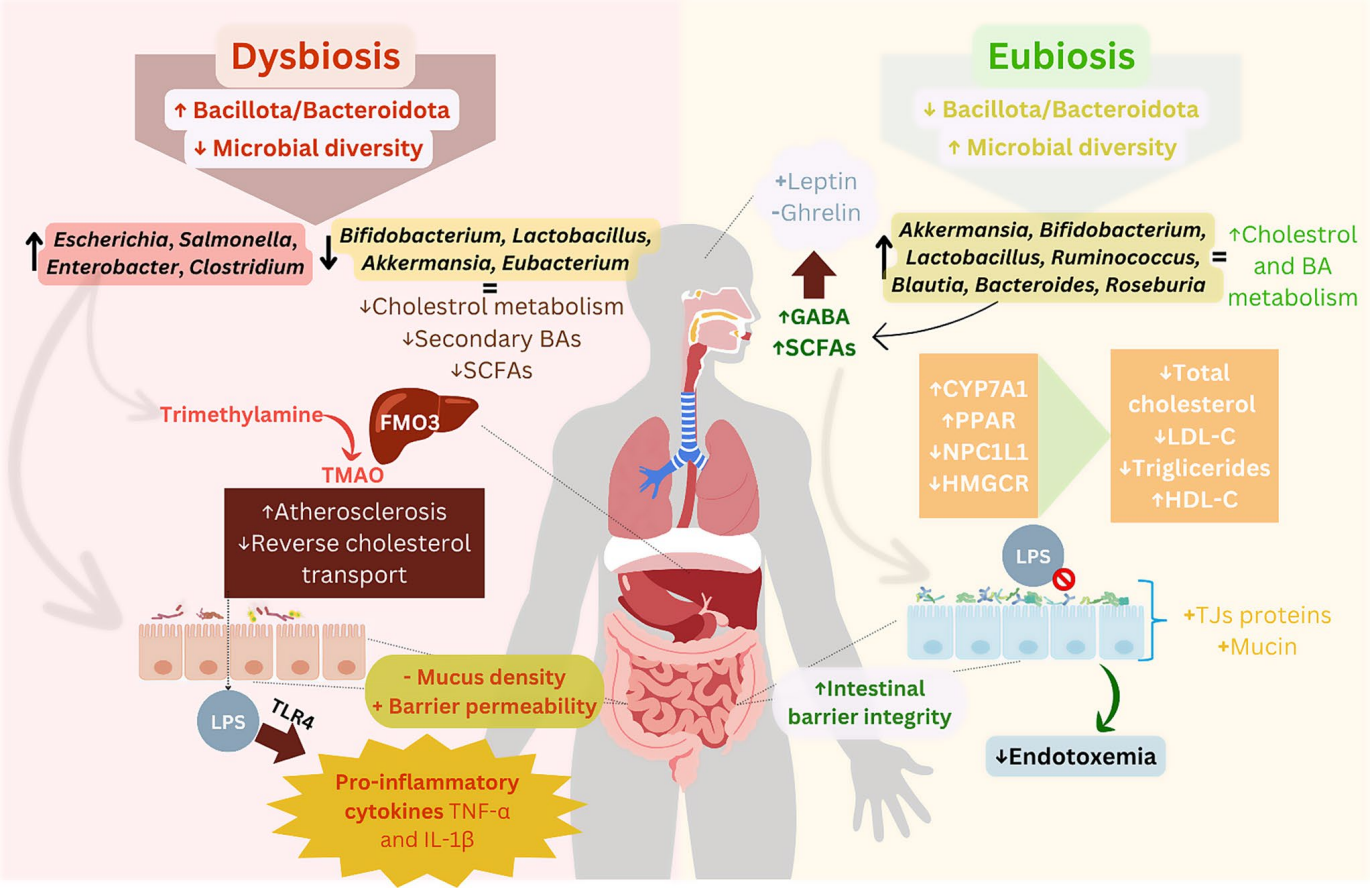
Status of gut microbiota on cholesterol management: Dysbiosis, characterized by decreased microbial diversity and higher concentration of harmful bacteria, decreases cholesterol metabolism and production of SCFA, while increasing inflammation and accumulation of atherosclerotic plaque. Eubiosis state, with more balanced microbial composition, promotes release of metabolites that enhance expression of cholesterol-lowering enzymes and reduce endotoxemia. (arrows indicate variation of concentration or expression ↑ increase and ↓ decrease). BA, bile acid; CYP7A1, cholesterol 7α-hydroxylase; FMO3, flavin-containing monooxygenase 3; GABA, gamma-aminobutyric acid; HMGCR, 3-hydroxy-3-methylglutaryl coenzyme a reductase; IL, interleukin; LPS, lipopolysaccharide; NPC1L1, Niemann–Pick C1-like 1 protein; PPAR, peroxisome proliferator-activated receptor; SCFA, short-chain fatty acids; TJ, tight junction; TLR, toll-like receptor; TMAO, trimethylamine N-oxide; TNF, tumour necrosis factor. References [25–38]

Gut microbiota-host interactions play an essential role in health and disease. In a balanced state, this community of commensal, symbiotic and pathogenic microorganisms supports the host’s metabolic health. Conversely, disruption of the gut microbiota can increase the risk of disease onset and progression [25]. Although dysbiosis varies with individual’s enterotype and other factors [26], several biomarkers are transversal in hypercholesterolemia-induced dysbiotic subjects. Figure 1 displays key interconnections between gut microbiota and cholesterol, summarising events involved in cholesterol homeostasis in eubiotic conditions and how these pathways are disrupted in dysbiosis, leading to additional adverse health consequences.

Elevated cholesterol alters the intestinal environment and disrupts gut microbiota balance, creating a specific dysbiotic profile. In hypercholesterolemic individuals, gut microbiota composition shows enrichment of *Pseudomonadota* and an elevated ratio of *Bacillota*/*Bacteroidota* (B/B) and may exhibit increased abundances of pathobionts, such as *Enterobacter* spp, *Escherichia-Shigella* and *Clostridium* [27]. On the other hand, the relative abundances of *Akkermansia*, *Roseburia*, *Faecalibacterium* and *Bifidobacterium* are often reduced, decreasing SCFA production and metabolites with indirect cholesterol-lowering properties [28, 29]. The reduction of SCFAs, notably butyrate, can weaken gut barrier integrity and promote inflammation, both of which are key drivers of atherosclerosis progression and insulin resistance [30]. These taxa have also been associated with cholesterol conversion to coprostanol [31] and modulation of key enzymes in cholesterol metabolism, such as CYP7A1 and HMGCR expression [32]. In addition, dysbiosis- associated reductions in microbial diversity have been linked to higher TG and low-density lipoprotein (LDL) cholesterol levels [33].

Gut microbiota also regulates BA metabolism, promoting bile acid excretion through bile salt hydrolase (BSH)-positive bacteria [34]. Microbial deconjugation and dehydroxylation of primary BA into secondary BA play a crucial role in cholesterol homeostasis. Enhanced fecal BA excretion prevents cholesterol reabsorption in the intestine and can lower circulating cholesterol. Conversely, BAs shape gut microbial composition by simultaneously promoting growth of resistant taxa and inhibiting bile-sensitive bacteria [35]. Increased primary BAs in hypercholesterolemia may impair BA metabolism and excretion, thereby shifting gut microbiota composition. Furthermore, increases in pro-inflammatory bacteria (*e.g. Proteobacteria*, *Enterobacteriaceae* and *Desulfovibrio* sp.) together with reductions in gut-barrier-supporting bacteria (*e.g. Bifidobacterium*, *Faecalibacterium* and *Akkermansia*) can facilitate passage of microbial-derived lipopolysaccharides (LPS) into the bloodstream and amplify inflammation [36, 37]. Similarly, trimethylamine-N-oxide (TMAO), derived from microbial metabolite trimethylamine, is often overproduced in dysbiotic settings and has been associated with pro-inflammatory effects, cholesterol accumulation and increased risk of atherosclerosis [38]. 

**Fig. 2:**
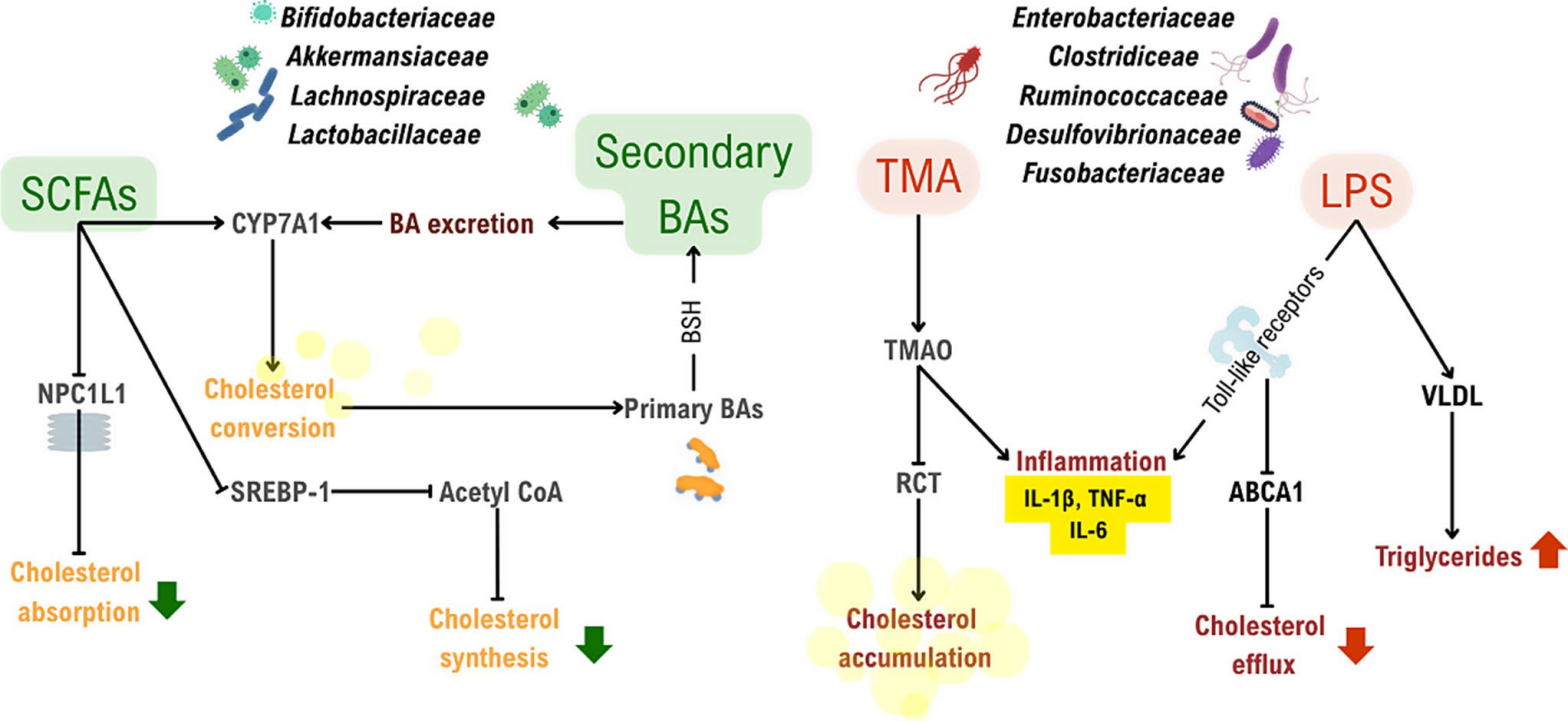
The role of gut microbiota metabolites in cholesterol metabolism: SCFAs inhibit NPC1L1 sterol transporter activity decreasing cholesterol absorption, promote cholesterol conversion to BAs by upregulation of CYP7A1 and down-regulate cholesterol synthesis through SREBP-1; secondary BAs produced by BSH-positive bacteria decrease BA pool size and promote CYP7A1 activity; TMA, converted to TMAO in the liver, triggers cytokine production and impairs RCT; LPS activates TLRs inducing inflammation and hindering cholesterol efflux via ABCA1 inhibition; LPS also stimulates VLDL release, increasing TG levels. Blunt arrows (┴) indicate inhibition, while sharp arrows (→) represent stimulation. ABCA1, ATP-binding cassette transporter A1; acetyl-CoA, acetyl coenzyme A; BA, bile acid; CYP7A1, cholesterol 7α-hydroxylase; IL, interleukin; LPS, lipopolysaccharide; NPC1L1, Niemann–Pick C1-like 1 protein; RCT, reverse cholesterol transport; SCFA, short-chain fatty acids; SREBP-1, sterol regulatory element-binding protein 1; TMAO, trimethylamine N-oxide; TNF, tumour necrosis factor; VLDL, very low-density lipoprotein. References [39–50]

SCFAs, BAs, TMAO and LPS are major gut microbiota-derived metabolites that directly interfere with cholesterol metabolism [39]. They are produced by specific gut taxa and affect cholesterol levels through distinct pathways (Fig. 2). SCFAs (acetate, butyrate and propionate) are produced by bacteria belonging to both low-abundant (*e.g. Verrucomicrobia*, *Actinomycetota*) and high-abundant (*e.g. Bacillota, Bacteroidota*) phyla. Commensal taxa within the families *Akkermansiaceae*, *Bifidobacteriaceae*, *Lachnospiraceae* and *Lactobacillaceae* are among the most significant SCFA producers [40]. These fatty acids act as signalling molecules, upregulating or inhibiting gene expression, thereby modulating multiple cholesterol-related pathways. Circulating SCFA levels have been linked to inhibition of cholesterol transport proteins (NPC1L1) [41] and suppression of sterol regulatory element-binding protein 1 (*SREBP-1*) gene expression, reducing substrate supply for cholesterol synthesis [42]. Moreover, CYP7A1, the rate-limiting enzyme that converts cholesterol to primary BAs, can also be up-regulated by SCFAs. Likewise, gut microbiota’s ability to reduce BA pool size through secondary BAs production and subsequent fecal excretion can trigger CYP7A1 activity (Fig. 2). BSH-positive bacteria, which deconjugate primary BAs, are commonly found in the genera *Bifidobacterium*, *Bacteroides*, and *Lactobacillus* as well as several members of the *Lachnospiraceae* family [43, 44].

TMAO and LPS are key contributors to chronic inflammation and elevated cholesterol and TG levels, therefore considered negative biomarkers of gut and cardiovascular health. As essential components of the cell wall of Gram-negative bacteria, LPS are produced by pathobionts such as *Desulfovibrio*, *Fusobacterium*, as well as several species of the *Enterobacteriaceae* family [45]. Increased LPS promotes the release of interleukins IL-1β, IL-6 and IL-18 via toll-like receptor 4 (TLR4) activation [46]. Collectively, TLR signalling can also suppress macrophage ATP-binding cassette transporter A1 (ABCA1) expression, thereby inhibiting cholesterol efflux [47]. Similarly, TMAO activates nuclear factor kappa B (NF-κB), triggering pro-inflammatory cytokine production [48]. Clinical trials have reported that TMAO impairs reverse cholesterol transport (RCT), increasing LDL-C and decreasing high-density lipoprotein (HDL-C) in vivo [38, 48]. Derived from dietary choline and carnitine, TMAO levels have been positively associated with several genera within *Bacillota* phylum, *e.g. Oscillibacter*, *Clostridium,* and *Romboutsia*, as well as *Pseudomonadota* phylum, notably *Klebsiella* spp. [49, 50].

## Hypocholesterolemic agents’ modulatory activity on gut microbiota

### NPC1L1 blockers

#### Ezetimibe

Ezetimibe is a synthetic cholesterol-lowering medication that blocks the sterol transporter NPC1L1 activity by occluding its transporter tunnel, preventing cholesterol entry into epithelial cells [51, 52]. This process decreases both endogenous and dietary cholesterol’s absorption leading to a significant increase in fecal excretion, resulting in cholesterol-lowering effect [53].

The interaction between ezetimibe and gut microbial composition appears to be limited to a few residual changes (Table 1). For instance, using normocholesterolemic mice, Catry et al. [54] did not observe any other significant change but the caecal increase of *Lactobacillus* spp. This shift in *Lactobacillus* abundance, however, was negatively correlated with *NPC1L1*, *SREBP* and *HMGCR* expression, and positively associated with CYP7A1 precursor, indicating the influence of this bacterial taxon in hypocholesterolemic effect. In fact, *Lactobacillus* species can mediate expression of genes related to cholesterol absorption, conversion and synthesis [32]. Nonetheless, a more recent study revealed that ezetimibe intervention caused a significant impact on hypercholesterolemic mice gut microbiota, reporting a decrease in α-diversity and relative abundances of phylum *Pseudomonadota* and genus *Desulfovibrio*, as well as increase in *Bacteroides* [56]. Ezetimibe treatment did not affect β-diversity or levels of health-promoting metabolites like SCFA. *Desulfovibrio* is commonly recognized as a pathobiont, observing increased abundances of this genus in inflammatory bowel disease and metabolic syndrome dysbiosis settings [63]. However, in a dyslipidaemia setting, Jin et al. [56] found that *Desulfovibrio* was negatively correlated with lipid markers (triglycerides and HDL-C), suggesting commensal status of this taxa within this context. Furthermore, a clinical trial of overweight adults reported adverse effect of ezetimibe therapy on gut microbiota, which might be related to its cholesterol-lowering mechanism of action. Reduced cholesterol absorption and concomitant accumulation in the colon resulted in a slight increase of the pathogenic genus *Fusobacterium* [55]. In that study, *Fusobacteriota* were positively correlated with aspartate transferase (AST) and negatively correlated with butyric acid, meaning that potential liver inflammation and reduced levels of SCFA were related to gut microbiota alterations brought by ezetimibe intervention.

**Table 1:** NPC1L1 blockers gut microbiota modulation

**Intervention compound** (dosage)	Model	Gut microbiota modulation			Outcome (metabolites, gene expression, metabolic pathways)	Reference
		Diversity	Phylum/Family	Genus/Species		
**Ezetimibe** (0.021% (w/w) , 1 week)	Standard diet mice (*n* = 32)	-	-	↔ *Bacteroides*-*Prevotella* spp., *Bifidobacterium* spp. ↑*Lactobacillus* spp.	↓*NPC1L1* ↑*SREBP-2*	[54]
**Ezetimibe** (10 mg/day, 12 weeks)	Overweight patients with dyslipidaemia (*n* = 26)	≠ β-diversity ↔ α-diversity	No significant changes	↑*Fusobacterium*	↔SCFA	[55]
**Ezetimibe** (10 mg/kg/day, 8 weeks)	HFD mice (*n* = 80)	↓Chao1 ↔Shannon = β-diversity	↓*Pseudomonadota*	↓*Desulfovibrio* ↑*Bacteroides*	↔Total SCFA ↓butyric acid	[56]
**PS** 95% purity (100 mg/kg/day, 8 weeks)	HFD mice (*n* = 18)	≠ β-diversity	-	↓*Allobaculum*, *Lactobacillus*, *Lactococcus*, *Streptococcus*, *Bacillus* and *Lactobacillus vaginalis*.	↓CYP7A1, FGFR4 ↑FXR-SHP pathway	[57]
**PS-enriched beverage** (2 g/250 mL, 48 h fermentation)	In vitro (feces from healthy donors, *n* = 5)	↔Shannon ↔Chao1 ≠β-diversity	↓*Erysipelotrichaceae*	↓*Allobaculum* ↑*Eubacterium halii*	↓Sitosterol, Stigmasterol	[58]
**Lotus seed powder PS extract** 62% purity (600 mg/kg, 5 weeks)	HCD mice (*n* = 40)	↓Shannon ↓Chao1 =β-diversity	↓*Bacillota*, *Verrucomicrobiota*, *Pseudomonadota* ↑*Bacteroidota*, *Actinobacteria*	↑*Yaniella*, *Sporosarcina*, *Oceanisphaera*, *Allobaculum*, *Virgibacillus* ↓*Bifidobacterium*, *Turicibacter*, *Akkermansia*, *Bilophila*	↓*NPC1L1*, ALT, AST	[59]
**Beta-sitosterol** (400 mg/kg/day, 8 weeks)	*ApoE*^*−/−*^ High Choline Diet mice (*n* = 24)	↑Shannon ↑Chao1 ≠β-diversity	↑*Actinobacteria*, *Bacteroidotes*, *Desulfobacterota*, *Bacillota* ↓*Pseudomonadota*, *Verrucomicrobiota*	↓*Klebsiella*, *Clostridioides*, *Desulfovibrio*, *Prevotella* ↑*Weissella*, *Eubacterium*, *Lactobacillus*, *Butyricioccus*	↓TMA, FMO3, TMAO, TNF-α, IL-6	[60]
**PS esters** (400 mg/kg, 24 days)	Cereal-based diet mice (*n* = 34)	↑Shannon ≠β-diversity	↓*Bacteroidota, Muribaculaceae* ↑*Verrucomicrobiota*	↑*Akkermansia*	↓CXCL1, CXCL2 ↑IgA	[61]
**PS esters** (0.05–0.1 g/100 g BW, 12 weeks)	NAFLD mice HFD (*n* = 24)	↑Shannon ↔Chao1 ≠β-diversity	↓*Verrucomicrobiota* ↑*Bacteroidota*, *Bacillota*, *Pseudomonadota*	↑*Anaerostipes, Adlercreutzia* ↓*Faecalibacterium, Phascolarctobacterium, Akkermansia*	↑isobutyric, valeric, isovaleric acid ↑TJs proteins	[62]

ALT, alanine transaminase; ApoE, apolipoprotein E; AST, aspartate transaminase; BW, body weight; CXCL, chemokine (C-X-C motif) ligand; CYP7A1, cholesterol 7α-hydroxylase; FGFR4, fibroblast growth factor receptor 4; FMO3, flavin-containing monooxygenase 3; FXR–SHP, farnesoid X receptor–small heterodimer partner; HFD, high-fat diet; IgA, immunoglobulin A; IL-6, interleukin 6; NAFLD, non-alcoholic fatty liver disease; NPC1L1, Niemann–Pick C1-like 1 protein; PS, phytosterol; SCFA, short-chain fatty acid; SREBP-2, sterol regulatory element-binding protein 2; TJ, tight junction; TMA, trimethylamine; TMAO, trimethylamine N-oxide; TNF, tumour necrosis factor. ↔ no variation, ↑ increase, ↓ decrease, = no distinction between groups (for beta-diversity), ≠significant differences between groups (beta-diversity)

#### Phytosterols

Phytosterols are triterpenes of similar structure and function to cholesterol, acting as important components of plant membranes [64]. PS are fat-soluble compounds abundant in several plants such as cereals, vegetables and nuts that have been shown to reduce cholesterol absorption and decrease in LDL-C levels [65, 66]. Absorption of dietary PS in the gut follows the same pathway as cholesterol, i.e. NPC1L1-mediated transport, followed by incorporation in micelles and subsequent absorption in enterocytes [67]. Therefore, the main proposed mechanism of PS cholesterol-lowering effect is through direct competition for intestinal absorption [8].

Impact of PS dietary supplementation on gut microbiota has been extensively studied in animal models, mostly conducted in hypercholesterolaemic mice (Table 1). Studies consistently report increased richness in α-diversity in addition to distinctive profiles of β-diversity between control and phytosterol-treated groups [57, 60]. At a phylum level, apart from conflicting reports regarding *Bacillota* relative abundance variation, a pattern of increasing abundances of *Bacteroidota* and decreasing *Pseudomonadota* and *Verrucomicrobiota* was observed [59, 60, 62], although Ye et al. [61] reported reduction of relative abundances of these bacterial taxa. Importantly, variation of *Pseudomonadota* and *Bacillota* phyla was negatively correlated with *NPC1L1* gene expression, thus linking PS-reduced cholesterol absorption to gut microbiota alterations [59].

Lower levels of taxonomy report recurring changes to *Lactobacillus*, *Akkermansia* and *Allobaculum* genera. Particularly, the *Allobaculum* genus was decreased after PS treatment in HFD mice [57] and also in vitro (human feces fermentation) [58], yet Liu et al. [59] study, using a comparable murine model, observed the opposite effect (increase proportion of *Allobaculum*). Nonetheless, this genus has been associated with hyperlipidaemia condition, and more interestingly a positive correlation between *Allobaculum* and expression of angiopoietin-like protein 4 (ANGPTL4) has been established [68], hinting at gut microbiota involvement in triglyceride-lowering effect of PS. ANGPTL4 inhibits activity of lipoprotein lipase, an enzyme that catalyses TG hydrolysis in muscle and adipose tissue [69]. Abundance of *Lactobacillus* was also consistently altered by PS interventions, a specific compositional modification positively correlated with CYP7A1 [57], cholesterol hydrolase, and negatively correlated with inflammatory mediators chemokine (C-X-C motif) ligand 1 and 2 (CXCL) [61]. Furthermore, the study of Wu et al. [60] reported increase of anti-inflammatory related taxa *Lactobacillus* and *Eubacterium* and decline in opportunistic pathogenic bacteria *Klebsiella* and *Desulfovibrio* by beta-sitosterol intervention, limiting production of trimethylamine (TMA) and subsequent conversion to TMAO.

Finally, *Akkermansia* genus shows a variable response to PS therapies, with evidence of both decrease [59, 62] and increase [61] in its relative abundance. *Akkermansia muciniphila* have been recently touted as a novel probiotic, active in restoration of intestinal barrier and as SCFA producing species [70]. In spite of this, increased isobutyric, valeric and isovaleric acid concentrations brought by phytosterol esters reported by Song et al. [62] might have been due to enhanced abundances of other SCFA-producing bacteria, probably belonging to genera *Adlercreutzia* and *Anaerostipes*. Consequently, release of SCFAs and concomitant lowering of colon pH might favour the growth of *Eubacterium halii* while reducing abundance of *Erysipelotrichaceae* [58].

### PPAR agonists (and partial agonists)

#### Fenofibrate

Fenofibrate is commonly used as an adjunct in dyslipidaemia management, providing pharmacological value via activation of PPAR α, a key transcription factor in lipid metabolism [71]. Acting as a PPAR α agonist, fenofibrate reduces triglycerides and LDL-C while increasing HDL-C levels through stimulation of gene expression of fatty acid and lipoprotein regulatory enzymes [72]. Fenofibrate promotes the β-oxidation pathway in the liver, decreasing fatty acid substrate availability for triglyceride and VLDL synthesis [73]. Also, PPAR α increases apolipoprotein expression, ApoAI and ApoAII, which are the main protein components of HDL [74].

The impact of fenofibrate monotherapy on gut microbiota has only been studied in vivo in animal models (HFD mice), as, to the best of our knowledge, no human clinical trials in this scope are currently available (Table 2). Regarding its gut microbiota modulatory activity, a slight increase in α-diversity was observed, despite not being relevant at a statistical level, while β-diversity showed distinct bacterial communities between control and treatment groups [75, 77]. These results directly oppose those found by Zhang et al. [7], where a decrease in diversity of microbial composition was observed. This study also reported that, at a phylum level, response was skewed toward *Bacillota* at the expense of *Bacteroidota* and *Actinomycetota*. The inverse scenario was observed in other studies [75–77], where a clear reduction of *Bacillota* and significant increase of *Bacteroidota* resulted in a lower B/B ratio. The discrepancy in these results may arise from the animal model used (*ob/ob* mice vs C57BL/6 mice) or treatment period (13 weeks vs 4 weeks).

**Table 2:** PPAR partial and full agonists gut microbiota modulation

**Intervention compound** (dosage)	Model	Gut microbiota modulation			Outcome (metabolites, gene expression, metabolic pathways)	Reference
		Diversity	Phylum/Family	Genus/Species		
**Fenofibrate** (20 mg/kg/day, 13 weeks)	*Ob/ob* HFD mice (*n* = 16)	↔Shannon = β-diversity	↑*Bacillota*, *Lachnospiraceae*, *Ruminococcaceae* ↓*Bacteroidota*, *Actinobacteria, Erysipelotrichales*	↓*Allobaculum* ↑*Clostridium*	↑PPARα, LDLR, SREBP-1c	[7]
**Fenofibrate** (0.1% w/w, 1 month)	HFD mice (*n* = 50)	↔Shannon ↔Simpson ≠β-diversity	↑*Bacteroidota*, *Porphyromonadaceae* ↓*Pseudomonadota*, *Bacillota*, *Desulfovibrionaceae, Lachnospiraceae, Ruminococcacaea*	↑*Alloprevotella, Bifidobacterium* ↓*Acetatifactor*, *Flavonifractor*, *Oscillibacter*, *Clostridium coccoides*	↑Total SCFA, acetate, propionate, butyrate ↓LPS	[75]
**Fenofibrate** (50 mg/kg/day, 9 weeks)	HFD mice (*n* = 18)	-	-	↑*Alistipes, Mycoplasma*, *Bacteroides ovatus, Bifidobacterium animalis, Bacteroides intestinalis, Lactobacillus reuteri* ↓*L. johnsonii, Lachnoclostridium* sp	↑UFA, indole ↓GT51	[76]
**Fenofibrate** (4 weeks)	HFD NAFLD mice (*n* = 30)	↔Shannon ↑Chao1 ≠β-diversity	↑*Bacteroidota*, *Verrumicrobiota*, *Muribaculaceae* ↓*Bacillota*, *Actinobacteria*	↑*Faecalibaculum*, *Akkermansia* ↓*Clostridium*, *Turicibacte*r, *Bifidobacterium*	-	[77]
**Curcumin** (0.1% (w/w), 12 weeks)	HFD hamsters (*n* = 16)	↓Shannon ↑Simpson ≠β-diversity	↓*Bacillota*, *Tenericutes* ↑*Bacteroidota*	↑*Bacteroides*, *Faecalibaculum*, *Phascolarctobacterium*, *Parasutterella* ↓*Lachnospiraceae, Oscillibacter*	↑CYP7A1 ↓NPC1L1	[78]
**Curcumin** (0.2% (w/w), 10 weeks)	HFD obese mice (*n* = 30)	↑Shannon ↑Chao1 ≠β-diversity	↓*Bacillota*, *Lachnospiraceae*, *Ruminococcaceae*, *Erysipelotrichaceae* ↑*Bacteroidota*, *Bacteroidaceae*, *Prevotellaceae*	↑*Bacteroides, Akkermansia, Parabacteroides, Alistipes, Alloprevotella* ↓*Desulfovibrio*, *Lactobacillus*	↓LPS, *SREBP-1c* ↑*PPARα*, caecal, colonic total SCFA	[79]
**Curcumin** (0.4% (w/w), 14 weeks)	HFD mice (*n* = 20)	↓Shannon ≠β-diversity	↓*Verrucomicrobiota*, *Ruminococcaceae*, *Burkholderiaceae* ↔*Bacillota*, *Bacteroidota*	↑*Lactococcus, Parasutterella*, *Turicibacter*	↔TG, leptin, FFA	[80]
**Quercetin** (100 µg/day, 12 weeks)	LDLR^−/−^ Mice HFD (*n* = 24)	↑Shannon ↑Chao1	↑*Actinobacteria*, *Bacteroidota* ↓*Bacillota*	↑*Bacteroides*, *Parabacteroides*, *Ruminococcus* ↓*Lactobacillus*	↑Caecal coprostanol ↓Total BAs, atherogenic LPC 18:1	[81]
**Quercetin** (100 mg/kg/d, 12 weeks)	*ApoE*^*−/−*^ HCD mice (*n* = 18)	-	No significant changes	↓*Phascolarctobacterium, Anaerovibrio*	↑primary BAs biosynthesis, cholesterol metabolism ↓TNF-α, IL-6	[82]
**Quercetin** (0.05%, 6 weeks)	HFD mice (*n* = 24)	↔Shannon ≠β-diversity	↓*Actinobacteria*, *Bacillota*, *Erysipelotrichaceae* ↑*Bacteroidota, Akkermansiaceae*, *Eggerthellaceae*	↑*Akkermansia*, *Bacteroides*, *Romboutsia*	↑propionic acid, ghrelin ↓Insulin, leptin, glucagon	[83]

ApoE, apolipoprotein E; BA, bile acid; CYP7A1, cholesterol 7α-hydroxylase; FFA, free fatty acids; GT51, glycosyltransferase family 51; HCD, high-cholesterol diet; HFD, high-fat diet; IL-6, interleukin 6; LDLR, low-density lipoprotein receptor; LPS, lipopolysaccharide; LPC, lysophosphatidylcholine; NAFLD, non-alcoholic fatty liver disease; NPC1L1, Niemann–Pick C1-like 1 protein; PPAR, peroxisome proliferator-activated receptor; SCFA, short-chain fatty acid; SREBP, sterol regulatory element-binding protein; TG, triglycerides; TNF, tumour necrosis factor; UFA, unsaturated fatty acids. ↔ no variation, ↑ increase, ↓ decrease, = no distinction between groups (for beta-diversity), ≠significant differences between groups (beta-diversity)

Administration of fenofibrate in HFD mice altered relative abundances of commensal and probiotic bacteria such as *Bacteroides ovatus*, *Bifidobacterium animalis*, and *Lactobacillus reuteri*. This variation at the species level was negatively correlated with LDL, total cholesterol (TC) and TG, and positively correlated with HDL-C and biosynthesis of unsaturated fatty acids [76]. Similarly, reported increases in abundances of *Alloprevotella* and *Bifidobacterium* following fenofibrate treatment were positively correlated with fecal SCFA, while LPS-associated bacteria *Acetatifactor, Flavonifractor* and *Oscillibacter* were decreased [75]. Altogether, these results point to a potentially positive impact on gut microbiota of fenofibrate treatment, partially reverting HFD induced dysbiosis, enhancing fenofibrate efficacy in cholesterol-lowering and mitigating inflammation brought by LPS-producing bacteria.

#### Curcumin

Curcumin is the main active compound of turmeric, a popular spice derived from the *Curcuma longa* rhizome [84]. This lipophilic polyphenol possesses several health benefits, but due to its poor bioavailability, its isolated consumption has had mixed results [85]. The cholesterol-lowering effect of curcumin supplementation is still controversial, with numerous clinical trials reviewed in meta-analysis displaying inconclusive results [86, 87]. However, scientific evidence supports curcumin‘s effect on triglycerides [88] and upregulation of LDL receptor (LDLR) protein expression [89]. In fact, some authors have reported a reduction of triglycerides in curcumin-treated subjects, despite, controversially, not reporting any significant changes to cholesterol [88, 90]. This effect could be due to curcumin’s multiple target interactions, with reported influence on PPAR-α [91] and SREBP-1c [92] expression, which affect pathways of triglyceride synthesis and catabolism [93, 94].

Regarding curcumin’s impact on gut microbiota, despite its prebiotic activity [95], reduction in microbial richness and diversity has been consistently reported in HFD dysbiotic mice [78], normal microbiota mice [96], and even mildly dyspeptic human subjects [97], showcasing instead, curcumin’s broad-spectrum antibacterial activity [98]. In fact, interactions between gut microbiota and curcumin present a bidirectional relation. In addition to a modulatory effect on gut microbiota, curcumin’s microbial metabolization and concomitant release of pharmacologically active metabolites increase its biological effects [99]. Curcumin administration affects gut microbiota community in a potentially beneficial manner, attenuating abundances of dysbiosis-elevated *Bacillota* phylum with parallel increase of *Bacteroidota*, decreasing B/B ratio [78, 79] (Table 2). At a genus level, curcumin intervention in HFD mice promoted a shift towards health-associated bacteria such as *Bacteroides*, *Akkermansia*, *Alistipes* and *Lactococcus*, observing a reduction in genera belonging to bacterial families *Desulfovibrionaceae*, *Ruminococcaceae* and *Lachnospiraceae* [78–80], results aligned with trials on normal diet rats [96].

Supporting the cholesterol-lowering effect of curcumin, microbial community alterations showed a positive correlation between *Bacteroides* and CYP7A1 liver expression. Several negative associations were also observed: *Akkermansia* and *Alistipes* with serum cholesterol and TG levels; *Desulfovibrio* with LPS endotoxin; and *Ruminococcaceae* with alanine transaminase (ALT), TC and TG levels [78, 79]. Furthermore, some articles have mentioned an increase of probiotic bacteria *Bifidobacterium* and *Lactobacillus* by curcumin intervention [100, 101], yet, despite supporting activity and growth of probiotic *Bifidobacterium* species [102], no conclusive evidence regarding actual growth stimulation of this genus by curcumin was found. In fact, clinical trials evaluating gut microbiota modulation by curcumin display a very heterogeneous response, with few similarities among intervention groups, e.g. Peterson et al. [103] reported that the only observable pattern in response to curcumin treatment was a decrease in relative abundances of *Blautia* spp. and *Ruminococcus* spp. Nevertheless, gut microbiota involvement in enhancement of curcumin health benefits was confirmed by the recent research of Luo et al. [104], where antibiotic-treated mice revealed a significantly lower level of curcumin deconjugation and concomitant production of active metabolites as opposed to normal microbiota mice.

#### Quercetin

Quercetin belongs to the class of flavonols, a subclass of flavonoids and is one of the most abundant phenolic compounds, widely distributed in plants [105]. Its use as a natural bioactive agent provides several health benefits, among others anti-inflammatory, antioxidant, hypotensive and hypolipidemic activities [106, 107]. The efficacy of quercetin as a cholesterol-lowering therapy is still inconclusive, while some clinical trials have observed significant reduction in LDL-C and TC levels [108], others only report a significant effect (decrease) on HDL-C serum levels [109, 110]. This bioactive compound, however, may enhance RCT and protect against the development of atherosclerosis [111]. Quercetin is able to induce expression of PPARγ/LXRα, which in turn is involved in HDL metabolic regulation, thus reducing risk of atherogenesis [12]. Moreover, despite contradictory reports, it has also been observed that quercetin treatment can enhance CYP7A1 activity [109].

Regarding its modulatory effect on gut microbiota, like other polyphenolic compounds, prebiotic and antimicrobial activity of quercetin might be the underlying cause of observed effects [110]. Quercetin supplementation can attenuate dysbiosis induced either by antibiotics [112], HFD [113], or leptin deficiency [114], exhibiting higher richness and diversity indexes of microbial composition. Most studies also show a decline in *Bacillota* and *Pseudomonadota* phyla with an increase of *Bacteroidota* and *Actinomycetota*, resulting in B/B ratio decrease [81, 83] (Table 2). At a genus level, quercetin intervention in dysbiotic animal models showed a significant decrease in common pathogenic taxa, *Escherichia* and *Shigella* [114], and parallel increase of commonly health-associated genera such as *Bacteroides* and *Akkermansia* [81, 83]. Accordingly, bacterial strains of *Bacteroides* have been shown to be responsible for quercetin transformation into active metabolites [115].

Related to the observed cholesterol-lowering activity of quercetin, Wu et al. [82] reported increased cholesterol uptake associated with primary bile acid biosynthesis in the quercetin intervention group, as well as higher concentrations of gut SCFA, results aligned with the study of Tan et al. [83]. In that study, the treated group exhibited increased values of propionic acid and CYP7A1 enzyme expression. Earlier, Zhang et al. [109] already suggested that quercetin-rich diet could upregulate CYP7A1 expression in rats, lowering cholesterol levels. Considering that an increase in SCFA circulating levels contributes to hepatic CYP7A1 activation, this could help explain quercetin’s cholesterol-lowering effect, via increased liver uptake for primary BA synthesis. Moreover, established positive correlations between *Bacillota* phylum and cholesterol, as well as *Actinomycetota* phylum and coprostanol (main cholesterol metabolic end product), reinforce gut microbiota’s role in cholesterol management ability of quercetin [81].

### HMGCR inhibitors

#### Red yeast rice

RYR is a natural product of Chinese origin, produced by fermenting cooked rice with *Monascus purpureus* mold, which results in a pigmented reddish, purple product [116]. RYR contains a series of bioactive compounds, the most relevant of which is monacolin K, structurally identical to lovastatin [117]. Monacolin K mechanism of action derives from its structural similarities to HMGCR, exerting its effect through competitive inhibition of this rate-limiting enzyme in cholesterol synthesis [9].

Reports on RYR’s influence on gut microbiota are scarce, and this interaction has only been assessed in murine models (Table 3). This supplement promotes structural changes at a high taxonomic level, through alterations in abundances of *Bacillota*, *Bacteroidota*, *Verrucomicrobiota* and *Mycoplasmatota* phyla [118, 119]. RYR intervention decreased B/B ratio as well as α-diversity, while β-diversity analysis showed significant changes in microbial communities [119]. At the family level, gut microbiota composition of subjects revealed consistent modifications in *Lachnospiraceae* and *Bacteroidaceae* abundances. Yang et al. [118] found that a decrease in abundance of *Lachnospiraceae* species was correlated to ameliorating effects on metabolic disorders. Yet, gut microbiota modulation by monascuspiloin and *Monascus* red pigment supplementation reported both negative [128] and positive [129] correlations of *Lachnospiraceae* operational taxonomic units abundance and cholesterol levels. The *Lachnospiraceae* genus richness may help explain these paradoxical results, as its abundance is simultaneously correlated with different diseases and production of beneficial metabolites [130]. For example, *Dorea* is a genus belonging to *Lachnospiraceae* family that is considered an obesity biomarker, associated with several cardiovascular risk factors [131]. However, bacterial genera like *Roseburia* and *Blautia*, which are known SCFA producing bacteria [132] are also grouped in the *Lachnospiraceae* family. Nevertheless, RYR treatment also promoted an increase in *Bacteroides* species and reduction of *Flavonifractor*, the latter being positively correlated to LDL-C and TC [119].

**Table 3:** HMGCR inhibitors gut microbiota modulation

**Intervention compound** (dosage)	Model	Gut microbiota modulation			Outcome (metabolites, gene expression, metabolic pathways)	Reference
		Diversity	Phylum/Family	Genus/Species		
**RYR** (6 mg/kg/d, 8 weeks)	High Fat diet SD rats (*n* = 50)	-	↓*Bacillota*, *Lactobacillaceae*, *Lachnospiraceae* ↑*Bacteroidota*, *Verrucomicrobiota, Prevotellaceae*	-	↑*CYP7A1* ↓*LPIN*	[118]
**RYR** (0.34 g/kg/day, 12 weeks)	*Apoe*^*−/−*^ HFD mice	↓Shannon ≠β-diversity	↓*Bacillota*, *Rikenellaceae* ↑*Tenericutes*, *Anaeroplasmataceae*, *Bacteroidaceae*	↑*Anaeroplasma*, *Bacteroides* ↓*Alistipes*, *Barnesiella*, *Flavonifractor*	↓HMGCR ↓TNF-α, IL-1β, TLR2, TLR4	[119]
**Genistein** (50 mg/day, 2 months)	Obese patients (*n* = 45)	↑Shannon ≠β-diversity	↑*Verrucomicrobiota*, *Cyanobacteria*, *Deferribacteres*, *Tenericutes* ↓*Bacillota*	↑*Paraprevotella*, *Suterella*, *Anaeroplasma*, *Oscillospira, Akkermansia muciniphila*	↓Metabolic endotoxemia ↑FFA, AA, secondary BAs	[120]
**Genistein** (3 mg/kg/d BW, 6 months)	HFD mice (*n* = 24)	↑α-diversity	↓*Bacteroidota*, *Bacteroidaceae* ↑*Bacillota*, *Verrucomicrobiota*, *Prevotellaceae*	↓*Bacteroides acidifaciens*, *Bacteroides uniformis* ↑*Faecalibacterium*, *Prevotella copri*, *Akkermansia muciniphila*	↓LPS, TLR4 ↑PGC-1α	[121]
**Daidzein** (0.6%, 16 weeks)	HFD mice (*n* = 24)	↑Shannon ↑Chao1 ≠β-diversity	↓*Bacillota*, *Actinobacteria*, *Pseudomonadota*	↑*Bifidobacterium*, *Bacteroides*, *Enterorhabdus*	↑Equol, FAO mRNA, hepatic *SIRT1*, *HNF4α*	[122]
**Soy isoflavone** 98.55% purity (0–50 mg/mL, 2 weeks)	In vitro SHIME	≠β-diversity	↓*Pseudomonadota*, *Bacillota* ↑*Bacteroidota*	↓*Klebsiella*, *Pseudomonas*, *Escherichia-Shigella*, *Staphylococcus* ↑*Bifidobacterium*, *Bacteroides*, *Dorea*, *Lactobacillus*, *Weissella*	↑SCFAs, acetic, lactic acid	[123]
**Soy isoflavone** (10 mg/kg/d, 15 days)	Kunming mice (*n* = 12)	↑Faith’s PD =β-diversity	↓*Bacillota* ↑*Bacteroidota*	↑*Lactobacillus*, *Adlercreutzia*, *Coprococcus*, *Ruminococcus*, *Butyricicoccus*, *Desulfovibrio* ↓*Facklamia*, *Jeotgalicoccus*, *Morganella, Candidatus arthromitus*	↓Acetic, valeric, isobutyric, isovaleric, caproic acid ↑butyric, propionic acid	[124]
**EGCG** (400 mg/kg, 12 weeks)	HFD SD rats (*n* = 10)	↔Shannon ↔Chao1 =β-diversity	↑*Bacteroidota*	↑*Butyricimonas, Allobaculum, Desulfovibrio, Dorea, Blautia, Streptococcus*, *Prevotella*	↓NEFA, AST, ALT	[125]
**EGCG** (100 mg/kg BW, 14 weeks)	HFD mice (*n* = 24)	↓Shannon ↓Chao1 ↑Simpson ≠β-diversity	↓*Bacillota*, *Pseudomonadota*, *Deferribacteres*, *Ruminococcaceae, Lachnospiraceae, Erysipelotrichacea* ↑*Verrucomicrobiota*	↑*Bacteroides*, *Parasutterella, Akkermansia* ↓*Allobaculum, Roseburia*	↓TNF-α, IL-6, IL-1β	[126]
**EGCG** (100 mg/kg BW, 6 weeks)	Obese HFD mice (*n* = 25)	↔Shannon ↔Chao1 ≠β-diversity	↓*Bacillota* ↑*Bacteroidota*, *Muribaculaceae*	↑*Alloprevotella*, *Bacteroides* ↓*Blautia*, *Mucispirillum*, *Colidextribacter*	↑5-HTP, GABA ↓SCFAs	[127]

5-HTP, 5-hydroxytryptophan; AA, Amino acids; ALT, Alanine transaminase; ApoE, Apolipoprotein E; AST, Aspartate transaminase; BA, Bile acids; CYP7A1, Cholesterol 7α-hydroxylase; EGCG: epigallocatechin gallate; FAO, Hepatic fatty acid oxidation; FFA, Free fatty acids; GABA, Gamma-aminobutyric acid; HFD, High-fat diet; HMGCR, 3-hydroxy-3-methylglutaryl coenzyme A reductase; HNF4α, Hepatocyte nuclear factor 4α; IL, Interleukin; LPS, Lipopolysaccharide; NEFA, Non-esterified fatty acid; RYR, red yeast rice; PGC, PPARγ coactivator; SCFA, Short-chain fatty acids; SD, Sprague–Dawley; SHIME, Simulator of the human intestinal microbial ecosystem; SIRT1, Sirtuin 1; TLR, Toll-like receptor; TNF, Tumour necrosis factor. ↔ no variation, ↑ increase, ↓ decrease, = no distinction between groups (for beta-diversity), ≠ significant differences between groups (beta-diversity)

#### Soy isoflavones

Soy isoflavones are bioflavonoids derived from soy products, occurring mainly as glycosides genistein and daidzein [133]. Soy isoflavones exhibit numerous biological functions preventive of cardiovascular disease through regulation of cholesterol levels [134]. Soy isoflavones cholesterol-lowering mechanism is not clearly defined, as multiple pathways have been proposed. Interference of isoflavones with SREBP-2 maturation and the subsequent upregulation of LDLR expression have been identified as probable mechanisms [135]. Additionally, considering that SREBP-2 also acts as a major regulator of HMGCR transcription, isoflavones daidzein and genistein have also been identified as HMGCR inhibitors, blocking cholesterol biosynthesis [136]. Furthermore, soy isoflavones metabolization by intestinal microbiota may explain their biological activity. Many bacterial families are known to degrade this type of polyphenol, among others *Clostridiaceae*, *Eubacteriaceae*, *Eggerthellaceae* and *Coriobacteriaceae* [137].

Isoflavones fermentation in the colon actively regulates diversity and richness of gut microbiota, selectively promoting growth or inhibition of bacterial species [123]. Both genistein and daidzein, in a hypercholesterolemic context, managed to increase gut microbial diversity in adults with obesity [120] and HFD mice [122] (Table 3). However, at a phylum level, animal and human studies have reported disparate findings, with some authors observing a decrease in *Bacillota* and parallel increase in *Bacteroidota* [123, 124], while others reported the opposite scenario or no change in these phyla, observing a marked increase in *Verrucomicrobiota* and *Actinomycetota* [120, 121]. These heterogeneous shifts could be related to dysbiotic or eubiotic state of test subjects’ gut microbiota profile, as an increase in *Bacteroidota* phylum was mostly found in the eubiotic state, both in in vitro [123] and in vivo experiments [124]. Otherwise, this microbial modulation is characterized by a logical increase in soy isoflavones bacterial degraders that convert these compounds into equol, O-desmethylangolensin or 4-ethylphenol [137]. Known producers of these metabolites include species of lactic acid bacteria, *Lactobacillus* and *Bifidobacterium*, as well as other health-associated bacteria from genera *Eubacterium*, *Blautia* and *Adlercreutzia* [138]. Isoflavone treatments have therefore resulted in an increase in *Bifidobacterium*, *Lactobacillus*, *Bacteroides* and *Adlercreutzia* abundances while also reducing relative abundance of opportunistic pathogenic species of *Klebsiella*, *Pseudomonas* and *Staphylococcus* genera [122–124]. Decrease in pathobiont bacteria could be related to isoflavone’s or its microbial-derived metabolites’ antibacterial activity [139]. Contrarily, studies focused on dyslipidaemic subjects with induced dysbiosis observed a different shift in microbial composition after genistein treatment, highlighting the increase of *Akkermansia muciniphila* [120, 121]. Alterations to gut microbiota composition in these studies were positively correlated with SCFA production, and negatively correlated with LPS, effectively reducing metabolic endotoxemia.

#### Epigallocatechin gallate

EGCG, the main catechin present in the tea plant (*Camellia sinensis*), displays a broad spectrum of therapeutic effects relevant in prevention of cardiovascular diseases [140]. Clinical trials have demonstrated EGCG’s antilipidemic effect in various patient profiles, albeit with inconsistent lipid-biomarker results [141, 142]. Tea catechin has demonstrated ability to significantly reduce LDL-C and TC levels regardless of subject baseline value, although not showing any relevant effect in HDL-C or TG [143]. Multiple mechanisms of action have been proposed for EGCG’s cholesterol-lowering effect: inhibition of cholesterol absorption, suppression of HMGCR, and upregulation of LDLR. Kobayashi et al. [144] suggested that EGCG may decrease micellar solubility of cholesterol. Other studies have demonstrated that EGCG inhibits the activity of HMGCR through cofactor competitive binding while simultaneously upregulating CYP7A1 expression [10, 143]. More recently, it was reported that EGCG may also exert LDL-C-lowering effect through inhibition of proprotein convertase subtilisin/kexin type 9 (PCSK9) production and LDLR upregulation [145].

Gut microbiota modulation by EGCG has been explored in vitro, by human fecal fermentation and in vivo, using HFD-induced obese mice (Table 3). In the in vitro study, prebiotic-like activity of EGCG clearly steered its impact on microbiota’s composition, stimulating growth of *Bifidobacterium*, *Lactobacillus* and *Enterococcus*, while inhibiting potentially pathogenic bacteria *Fusobacterium*, *Enterobacteriaceae* and *Clostridium* [146, 147]. This EGCG’s microbiota modulation was also accompanied by relevant increases in total SCFA, driven by release of acetic and butyric acid. Conversely, animal studies present a distinct and more heterogenous EGCG’s microbial modulatory impact. For instance, similarly-designed studies have reported increase [148], decrease [126] and unaltered [125] gut microbiota diversity and richness indexes by EGCG treatment. Changes at phylum level, however, follow a more consistent pattern, with several authors reporting EGCG induced relative abundance of *Bacteroidota* in detriment to *Bacillota*, with corresponding decrease in B/B ratio [125, 127, 149]. In vivo research suggests that EGCG treatment may revert dysbiosis condition via gut microbiota’s composition modulation, promoting SCFA production and decreasing inflammatory biomarkers. Supporting this claim, Wen et al. [125] found that EGCG enhanced relative abundance of *Butyricimonas* and *Desulfovibrio*, butyrate and acetate-producing bacteria respectively, while Cheng et al. [149] reported an increase in other SCFA-producing taxa, *Faecalibacterium* and *Bifidobacterium*. Furthermore, EGCG treatment has promoted abundances of *Dorea* and a parallel decrease of *Blautia* and *Ruminococcus* [125, 126]. These latter genera have been positively associated with LDL-C, TC and TG levels, while the former are negatively correlated with AST and TC levels, providing circumstantial evidence of EGCG cholesterol-lowering and anti-inflammatory effect through gut microbiota modulation. These observations showcase that the pathobiont status of a certain taxa is dependent on gut health conditions as *Dorea*, here associated with health benefits may otherwise be correlated with obesity biomarkers [131], as previously mentioned in Sect. “Red yeast rice”. Opposing the previously reported observations, Unno et al. and Zhou et al. [127, 150] registered a reduction of SCFA after EGCG treatment, associated with a decrease in abundance of *Bifidobacterium*, *Clostridium* and an increase of *Blautia*. Finally, reported *Akkermansia muciniphila* enrichment in mice gut microbiota [126, 146] is likely related to this species’ ability to metabolize EGCG, competitively promoting its growth [151].

### Other natural and synthetic hypocholesterolemic agents

#### Bile acid sequestrants

BAS, such as cholestyramine and colesevelam, are positively charged indigestible resins that bind to BA and by forming insoluble complexes provide a reduction in BA enterohepatic circulation [152]. This process not only affects cholesterol absorption, by inhibiting lipid-solubilizing activity of BAs [153], but also stimulates cholesterol conversion to BAs by *de novo* synthesis in the liver [154].

Reported alterations in gut microbiota after BAS treatment in vivo follow a pattern correlated with changes in BA availability. By blocking BA absorption through sequestration in the intestine, BAS activity increases the concentration of primary BAs in the cecum and colon [155], thereby promoting gut microbiota alterations based on taxa’s bile tolerance or sensitivity. Decreasing levels of BAs favour growth of Gram-negative bacteria, including LPS-producing and pathobionts, whereas an increment in BAs shifts community towards Gram-positive bacteria, members of the *Bacillota* phylum, which have a genetic proclivity to tolerate and metabolize BAs [156]. In fact, besides Gram-stain grouping, BSH competence is also a predominant factor in gut microbiota response to BAS intervention. In that regard, the study by Fuchs et al. [157] revealed that colesevelam treatment enhanced relative abundance of the *Delta-Pseudomonadota* class, mainly within *Desulfovibronacae* family, and despite no significant change in the *Bacillota* phylum, an increase in *Lactobacillus* species was observed (both BSH-positive bacterial groups). Similar studies in hypercholesteremic models also observed changes in gut microbiota composition in BAS-treated group, documented by increment in Chao1 and Shannon α-diversity indexes, as well as a clear disparity in β-diversity between treatment and control groups (Table 4) [158, 159]. However, no such alterations were observed in humans, as microbial community diversity remained unchanged after BAS treatment [160]. Yet, similarities between the different in vivo models used can be found, i.e. cholestyramine treatment has uniformly increase *Lachnospiraceae* family abundance, such change was positively correlated with SCFA and secondary BAs excretion and negatively with IL-18 [159, 160]. However, Kusumoto et al. and Park et al. [158, 170]observed a different scenario, both reporting an increase in *Bacteroides* and *Clostridium* groups with concomitant increment of acetate and propionate cecum levels. Evidence suggests that BAS treatment enhances BSH-positive bacteria, such as those belonging to *Bacteroides* and *Lachnospiraceae* [44], which in turn increases production of SCFA and secondary BAs. Correlated to these gut microbiota-derived metabolites, an increase in mRNA expression of *CYP7A1* was reported [157], enhancing the cholesterol-lowering ability of BAS.

**Table 4:** Other cholesterol-lowering therapies gut microbiota modulation

**Intervention compound** (dosage)	Bioassay Model		Gut microbiota modulation					Outcome (metabolites, gene expression, metabolic pathways)	Reference
			Diversity		Phylum/Family		Genus/Species		
**Colesevelam** (2% (w/w), 8 weeks)	*Mdr2*^*−/−*^ mice (*n* = 14)		-		↑*Pseudomonadota*, *Desulfovibronacae*, *Lactobacillaceae*		↑*Lactobacillus*	↑BA excretion ↑GLP-1	[157]
**Cholestyramine** (2% (w/w), 8 weeks)	Sham cholecystectomy HFD mice (*n* = 60)		↔Shannon ↔Chao1 = β-diversity		↓*Lachnospiraceae* ↑*Bacteroidaceae*		↑*Bacteroides*, *Oscillospira, Olsenella* ↓*Desulfovibrio*	↑acetate, mucin-producing goblet cells	[158]
**Cholestyramine** (2% (w/w), 10 weeks)	Western diet mice (*n* = 15)		↑Shannon ≠ β-diversity		↓*Ruminococcaceae, Muribaculaceae* ↑*Lachnospiraceae*, *Turicibacteraceae*, *Clostridiaceae*		↑*Acetatifactor muris* ↓*Muribaculum intestinale*	↑GLP-1 precursor	[159]
**Cholestyramine** (16 g/day, 16 weeks)	Primary biliary cholangitis patients (*n* = 33)		↔Shannon = β-diversity		↑*Lachnospiraceae*		↑*Klebsiella pneumoniae* ↓*Roseburia intestinalis*	↓ALP, FGF19 ↑GLP-1, SCFA, secondary BAs excretion	[160]
**Barley β -glucan** 75% purity (4 weeks)	HCD rats (*n* = 48)		-		-		↑*Bifidobacterium* ↓*Lactobacillus*, *Bacteroides*-*Prevotella*	↑Total organic acids, SCFA	[161]
**Bread w/β -glucan** (3.4 g/100 g, 4 weeks)	Metabolic syndrome subjects (*n* = 43)		↓Shannon ↓Chao1 =β-diversity		↓*Coriobacteriaceae*, *Clostridiceae*, *Bacillota* ↑*Bacteroidota*		↑*Eubacterium rectale*, *Bifidobacterium bifidum*, *Akkermansia muciniphila* ↓*Clostridium leptum*	↑Propionic acid ↓Acetic acid	[162]
**HMW barley β-glucan** (3 g/d, 5 weeks)	Hypercholesterolemic subjects (*n* = 19)		↔Chao1 ↔Shannon ≠β-diversity		↑*Bacteroidota*, *Rikenellaceae* ↓*Bacillota*		↑ *Bacteroides*, *Prevotella* ↓*Dorea*, *Streptococcus*	↑glycerolipid metabolism, glycan degradation	[163]
**Oat β-glucan** 70% purity (1 g/kg BW, 24 weeks)	Western-diet mice		≠β-diversity		↑*Lachnospiraceae*, *Ruminococcaceae*		↓*Alistipes* ↑*Lactobacillus*	↑Total SCFAs, butyrate ↓*TLR4*, *TLR9*, ALT, AST	[164]
**Oat β-glucan** (3 g/day, 45 days)	Hypercholesterolemia subjects (*n* = 187)		↔α-diversity =β-diversity		↓*Sutterellaceae*		↓*Lactobacillus, Roseburia* ↑*Dialister, Butyrivibrio, Paraprevotella, Bifidobacterium*, *Akkermansia muciniphila, Faecalibacterium prausnitzii*	↑Acetic, propionic acid, carbohydrate esterases, glycosyltransferases	[165]
**β-glucan extract** *Lentinula edodes* (3.5 g/d, 8 weeks)	Hypercholesterolemic subjects (*n* = 52)		=phylogenetic diversity		↑*Ruminococcaceae*		↑*Bifidobacterium* ↓*Dorea, Coprococcus*	↔cytokine profile, oxLDL	[166]
**Berberine** (100 mg/kg/d, 10 days)	*Ob/ob* mice (*n* = 18)		-		↑*Lachnospiraceae*		↑*Enterobacter*, *Escherichia*−*Shigella*, *Akkermansia*, *Clostridium*, *Bacteroides*	↑Butyrate	[167]
**Berberine** (500 ppm, 12 weeks)	*Apc* ^min/+^ HFD mice (*n* = 15)		↓Shannon ≠β-diversity		↓*Verrucomicrobiota, Bacteroidota* ↑*Bacillota, Lachnospiraceae, Bacteroidaceae, Prevotellaceae*		↓*Akkermansia*, *Bacteroides* ↑*Bilophila*	-	[168]
**Berberine** (100 mg/kg/week, 12 weeks)	*ApoE*^*−/−*^ HFD mice (*n* = 32)		-		↑*Verrucomicrobiota*, *Bacillota* ↓*Pseudomonadota*, *Bacteroidota*		-	↓TMAO, FMO3, IL-6, *TNF-α*, *IL-1β*	[169]

ALP, Alkaline phosphatase; ALT, Alanine transaminase; ApoE, Apolipoprotein E; AST, Aspartate transaminase; BA, Bile acid; FGF19, Fibroblast growth factor 19; FMO3, Flavin-containing monooxygenase 3; Gcg, Glucagon; GLP-1, Glucagon-like peptide 1; HCD, High-cholesterol diet; HFD, High-fat diet; HMW, High molecular weight; IL, Interleukin; Mdr, Multidrug resistant; oxLDL, Oxidized low-density lipoprotein; SCFA, Short-chain fatty acids; TLR, Toll-like receptor; TMAO, Trimethylamine N-oxide; TNF, Tumour necrosis factor. ↔ no variation, ↑ increase, ↓ decrease, = no distinction between groups (for beta-diversity), ≠ significant differences between groups (beta-diversity)

#### Beta-glucans

The term “dietary fibre” is used to describe a large group of indigestible polysaccharides that display multiple beneficial roles, among others hypocholesteraemic effect [171]. β-glucans are well-known dietary fibres that display a cholesterol-lowering effect by several mechanisms: formation of glucan-biliary salt complexes, which increase BA excretion and promote cholesterol metabolism, or through increased viscosity of luminal contents that hinders cholesterol absorption [13]. β-glucans are also prebiotics, thereby inhibiting cholesterol biosynthesis through gut microbiota fermentation and release of SCFAs, as explained in Sect. “Overview of gut microbiota-cholesterol interactions”.

β-glucans are prebiotic polysaccharides that promote selective growth of gut taxa based on their genetic and enzymatic ability to degrade complex dietary fibres. Within the *Bacteroidota* phylum, *Bacteroides* and *Prevotella* genera are efficient fermenters of β-glucan due to a large repertoire of glycoside hydrolases expression encoded by their polysaccharide utilization loci (*PUL*)-rich genomes [172, 173]. Inversely, Gram-positive *Bacillota* with mostly absent *PUL* genes, are unable to degrade complex glycans structures such as β-glucans [174], despite notable exceptions, *Eubacterium rectale* [175] or *Blautia producta* [176]. Furthermore, β-glucans microbial-derived metabolites promote growth of other genera of bacteria, *e.g. Bifidobacterium* spp., which easily metabolizes oligosaccharides released during β-glucan fermentation by other gut bacteria [172].

Within a hypercholesterolemia-induced dysbiosis context, β-glucans supplementation has revealed a clear beneficial impact on the host’s gut microbiota, both in animal [161] and human trials [166]. β-glucans shift in gut microbial composition is mostly characterized by a decrease in B/B ratio, driven by increased abundances of *Bacteroides* and *Prevotella* and decline in population of *Dorea*, *Streptococcus*, and *Clostridium* [162, 163]. Regarding bacterial α/β diversity, minimal or no effect was observed in clinical trials [162, 166]. β-glucans also affect *Lactobacillus* genus but in a non-consistent manner, observing instances of increase [164] and decrease [161, 165] of its relative abundance in the host’s microbial community. This divergent outcome might be due to differences in β-glucan’s molecular weight (MW) used in these studies’ intervention groups. Low-MW β-glucan generally promotes *Lactobacillus* growth, while high-MW exhibit the opposite effect [177]. Growth of other common probiotics, specifically *Bifidobacterium* and *Akkermansia*, was reported after β-glucan intervention, attenuating pre-treatment dysbiosis condition in mild hypercholesterolemic patients [162, 165]. Unsurprisingly, these compositional changes were accompanied by an increase in acetate, propionate and butyrate, as modulation of gut microbiota tilted towards SCFA-producing bacteria [161, 164]. In summary, the cholesterol-lowering effect of these dietary fibres is influenced by gut microbiota modulation, displaying negative correlations between enhanced *Prevotella* and *Bifidobacterium* genus abundances and LDL-C, TG and TC levels [163, 165, 166].

#### Berberine

Berberine (BBR) is a natural alkaloid, a secondary metabolite extracted from the rhizome of *Coptis chinensis* Franch, extensively used in traditional Chinese medicine [178]. Berberine’s pharmacological and therapeutic potential is enhanced by its multiple cellular and molecular targets, affecting several metabolic processes via regulation of broad-spectrum signalling pathways [179]. This bioactive alkaloid affects several key targets of cholesterol metabolism in the intestine and liver, which translates into a substantial effect on cholesterol levels [180]. Berberine is able to reduce LDL-C levels through inhibition of PCSK9 expression via acceleration of hepatocyte nuclear factor (HNF1α) degradation, a mediator factor in PCSK9 transcription [181]. This cascade of events promotes upregulation of LDLR, increasing LDL uptake and causing its plasma level reduction [182]. RCT is also promoted by berberine through expression of ABCA1, a key mediator of cholesterol efflux into HDL particles [183]. Furthermore, BBR’s combined low bioavailability [184] and known antimicrobial effect [185] suggest indirect cholesterol-lowering properties through modulation of gut microbiota.

Sun et al. [186] found that berberine inhibited BSH activity of gut microbiota, leading to an increase in primary BAs, mainly taurocholic acid (TCA), a known agonist of intestinal Farnesoid X Receptor (FXR). The lipid-lowering effect observed may be related to FXR signalling activation, shown to improve hepatic triglycerides [187]. Additionally, Tian et al. [188] also observed that BBR’s intervention group exhibited a decrease in BSH-positive bacteria from *Bacillota* phylum, *Clostridium coccoides* and *leptum* groups, resulting in accumulation of TCA and activation of intestinal FXR. In hyperlipidaemic patients, cholesterol-lowering activity of BBR was influenced by the modulation of *Blautia* and *Alistipes* abundances., while no anti-lipidemic effect was observed in similarly treated pseudo-germfree mice [189].

Nonetheless, it has been hypothesized that berberine affects gut microbiota in an indiscriminate manner, reflected by mixed results of in vivo experiments. In fact, despite previously described positive interactions, BBR intervention in healthy mice researched by Yue et al. [190] observed decreased richness and diversity of microbial composition leading to dysbiosis. This study associated an increase in relative abundances of *Parabacteroides* and *Prevotella* and a decrease of caecal SCFA to berberine-induced mild diarrhoea observed in the treated group. Conversely, HFD mice treated with BBR reportedly exhibited growth of various SCFA-producing taxa, both pathobionts and commensal bacteria: *Enterobacter*, *Escherichia-Shigella*, *Akkermansia*, *Clostridium* and *Bacteroides* [167]. Unsurprisingly, this altered microbial composition led to an increase in butyrate and propionate exerting lipid-lowering effects. Nevertheless, the relatively brief period of treatment (10 days) in this research could be a confounding factor. Wang et al. [168] found that BBR promoted abundance of *Lachnospiraceae* family, reinforcing the notion that this alkaloid may consistently upregulate butyrate-producing bacteria. However, the same study reported decrease of *Akkermansia* and *Bacteroides* abundances, with overall decrease of gut microbiota α-diversity, contradicting previous studies. Finally, TMAO levels can also be affected through BBR’s gut microbiota modulation, which has been negatively correlated with expression of flavin-containing monooxygenase 3 (FMO3), enzyme catalyses conversion of trimethylamine to TMAO [169].

## Patterns of hypocholesterolemic agents’ microbiota modulation

All cholesterol-lowering agents reviewed in this study, both natural and synthetic, displayed gut microbiota modulatory activity. Intake of hypocholesterolemic agents induced differences in microbial community expressed via β-diversity analysis, as most studies reported dissimilar microbial profile between treated and non-treated groups, with the notable exception of BAS. Impact of cholesterol-lowering therapies on richness and evenness of microbial profile, measured by Chao1, Shannon and Simpson indices, displayed heterogeneous responses. Instances of increased (phytosterols, soy isoflavones, curcumin and 1uercetin), decreased (β-glucans, RYR, berberine, EGCG) or unaltered (ezetimibe, BAS, fenofibrate) α-diversity profiles were reported. This insight suggests overall community restructuring, shifting gut microbiota composition, occurs systematically, while not always translating into an increase in diversity. This heterogeneity implies that diversity metrics alone are insufficient to infer whether the intervention moves the microbiota toward a more eubiotic state and thus increases the pharmacological value of a specific hypocholesterolemic agent.

At a phylum level, elevated *Bacillota/Bacteroidota* ratio, often associated with dyslipidemia and hypercholesterolemia, was reduced by most compounds (except for ezetimibe and berberine), thus consistently counteracting hyperlipidemia-associated microbial signatures, even if at a high taxonomic level. Regarding low-abundant phyla modulation, apart from *Verrucomicrobiota*, which was increased by most compounds, other phyla were sparsely influenced with but only a few positive alterations to the *Actinomycetota* phylum and a decrease in abundances of *Pseudomonadota*, which mostly include pathobiont bacteria.

**Fig. 3:**
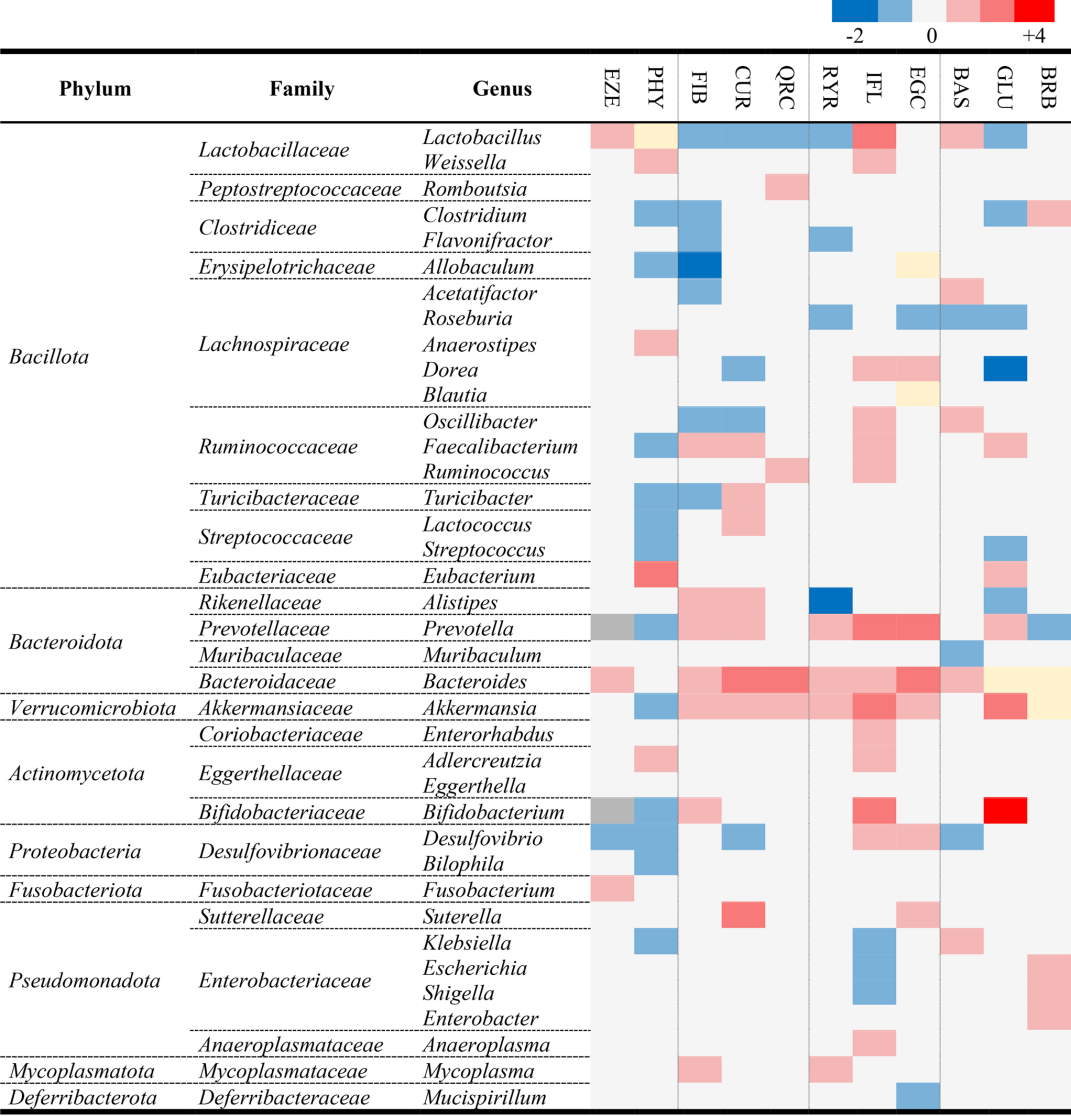
Heatmap of microbial taxa variation induced by hypocholesterolemic agents. Intensity of colour scaled by number of references, blue indicates decrease in abundance, red indicates increase. Light grey: no information about bacterial taxa variation, dark grey: no variation found, light yellow: conflicting data of bacterial taxa variation (net zero). BAS, bile acid sequestrants; BRB, berberine; CUR, curcumin; EGC, epigallocatechin gallate; EZE, ezetimibe; FIB, fibrates; GLU, beta-glucans; IFL, soy isoflavones; PHY, phytosterols; QRC, quercetin; RYR, red yeast rice

Modulatory activities at family and genus level are depicted in Figure 3 heatmap, which agglomerates data of hypocholesterolemic agents’ impact on gut microbiota expressed by bacteria taxa abundances’ variation. Discrimination of these agents by origin, either natural or synthetic, revealed no discernible patterns of modulatory activity. However, grouping these agents by molecular target, we may observe the following patterns: (i) NPC1L1 blockers or cholesterol absorption inhibitors such as ezetimibe and phytosterols promote increase of genera within *Lactobacillaceae* family and decrease *Desulfovibrio* genus belonging to *Desulfovibrionaceae* family; (ii) PPAR agonists (fenofibrate) and partial agonists (curcumin and quercetin) reduce *Lactobacillus* abundance, promote growth of *Bacteroides* and *Akkermansia* genera and modulate bacteria abundances within *Ruminococcaceae* family; (iii) HMGCR inhibitors, RYR, soy isoflavones and EGCG increase *Prevotella*, *Bacteroides* and *Akkermansia* genera abundances and modulate gut microbiota populations within *Lachnospiraceae* family. This modulatory profile shares a few similarities with those reported in synthetic statins (HMGCR inhibitor) interventions, which also revealed an increase in *Akkermansia* and *Bacteroides* genus [21, 191]. Concerning the remaining cholesterol-lowering compounds, BAS may influence abundances of BSH-positive bacteria *Lactobacillus*, *Bacteroides* and *Acetatifactor*. β-glucans modulatory activity discriminated based on microorganism proclivity to degrade glucans or glucan-derived oligosaccharides, thus promoting growth of *Prevotella*, *Eubacterium*, and strongly increase *Bifidobacterium* abundances, while these genera outcompete bacteria from *Lachnospiraceae* family, namely *Dorea* and *Roseburia*. Lastly, berberine’s conflicting data is expressed by its almost empty column in the heatmap (Fig. 3), as both negative and positive variations of the same bacteria taxa were found, thus discarding significant modulatory effects at the genus level.

Bioavailability and molecular target of the hypocholesterolaemic agent are key factors affecting degree and pattern of gut microbiota profile modulation. Clustering these compounds by mechanistic class allowed emergence of discernible patterns of modulatory activity based on dominant site of action. For instance, NPC1L1- related strategies showed limited microbiota effects as their extent of action is restricted to host transport processes. Contrarily, agents with broader exposure, such as those that affect overall BA availability (BAS and β-glucans) or reach the colon in appreciable amounts due to low bioavailability, are more likely to produce significant gut microbiota restructuring. In fact, low bioavailability of some of these cholesterol-lowering agents may translate into selective prebiotic activity, thus conditioning their gut microbiota modulatory activity to specific taxa with the ability to metabolize a particular compound. This appears to be the case in most polyphenol-based therapies (EGCG, curcumin, quercetin and berberine) as well as β-glucans, as their antimicrobial and/or prebiotic effect produces microbial response driven by ecological selection. Furthermore, microbial degradation of these compounds into bioactive metabolites may increase these agents’ hypocholesterolemic effect and overall therapeutic value.

**Fig. 4:**
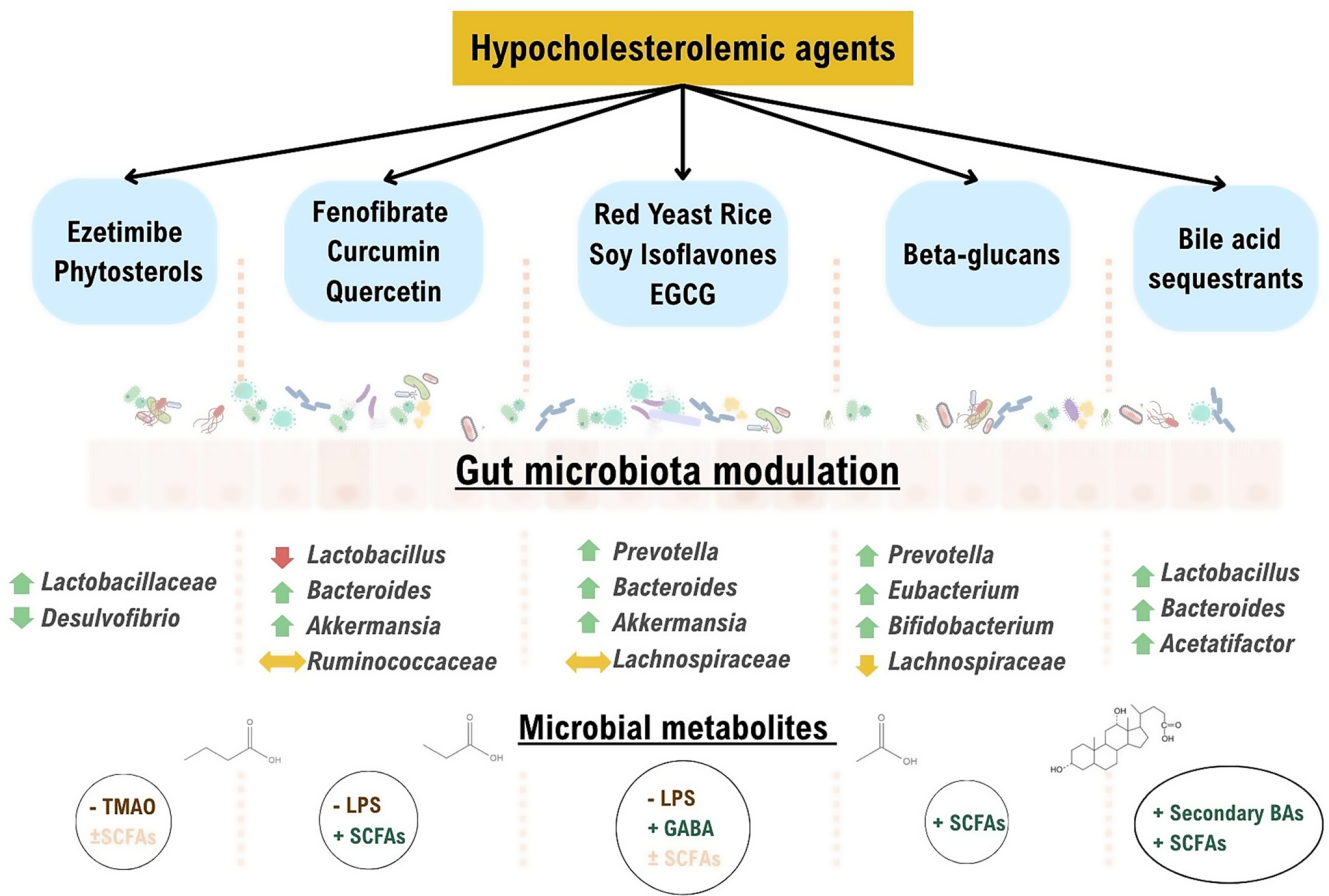
Modulatory activity of hypocholesterolemic agents on gut microbiota, involving alterations of main bacterial genera and families and correlated shifts in microbial metabolite production. BAs, bile acids; EGCG, epigallocatechin gallate; GABA, gamma-aminobutyric acid; LPS, lipopolysaccharide; SCFA, short-chain fatty acids; TMAO, trimethylamine N-oxide

As summarized in Fig. 4, anti-cholesterol interventions shifted gut microbiota composition towards anti-inflammatory bacteria, with concomitant release of health-inducing metabolites (SCFAs) with gut-barrier support functions, at the detriment of pathobiont abundances that are associated with inflammatory markers TMAO and LPS. Multiple statistically significant correlations between bacterial taxa abundances shifts, and lipid markers (TC, LDL, TG and HDL) or enzymes/gene expression related to cholesterol metabolism suggest that microbiota modulation of these compounds is involved in the cholesterol-lowering effect. Most treatments included in this review showcased relevant association between anti-cholesterol activity and microbial modulation, although causality between improved host lipid markers and microbial changes cannot be established. Yet, studies conducted on germ-free mice support this claim, as curcumin [104], berberine [189], bile acid resins [170], EGCG [192], and quercetin [193] administration observed a mitigated cholesterol-lowering effect in models with absent gut microbiota.

## Limitations

This study presents several limitations, mostly related to current gut microbiota research methodology and lack of replicable conditions. First, most eligible studies reviewed here were conducted in mice and other animal models, with experimental trials in humans representing but a fraction of the research on the impact of cholesterol-lowering therapies on gut microbiota. Second, different dosages and treatment periods applied within the same category of anti-cholesterol therapy hinder effective result comparison and pattern emergence. Overall heterogeneity between studies, originating from intervention duration, dosage, and in vivo models, as well as inter-individual specificity of gut microbiota, present significant limitations. To overcome these issues, more human studies focused on individual-specific, gut microbiota-induced physiological responses to medication are necessary.

## Conclusions

This review provided insights into how natural and synthetic cholesterol-lowering agents influence gut microbiota composition and diversity. Shifts in β-diversity and a decrease in *Bacillota*/*Bacteroidota* were the main hallmarks observed for several agents, whereas finer taxonomic evaluation often showed heterogeneous microbiota responses. Nevertheless, alongside improved lipid metabolism, these agents’ modulatory action often favoured SCFA-associated and anti-inflammatory bacteria and reduced LPS/TMAO-related taxa. Importantly, comparative analysis based on mechanistic class, molecular target and intestinal exposure/bioavailability revealed more informative patterns of gut microbiota modulation at a family and genus level. In contrast, categorizing agents as natural or synthetic provided no distinguishable microbial signature. Collectively, the evidence supports evaluating cholesterol management strategies not only by classical lipid markers but also by their capacity to reshape microbiota communities and their associated metabolic and inflammatory pathways. More mechanism-driven and potentially personalized therapeutic approaches should be the focus of future discussions and study designs, with bioavailability and molecular targets as key explanatory axes for medication-microbiota interactions.

## Data Availability

No datasets were generated or analysed during the current study.

## Abbreviations

5-HTP: 5-hydroxytryptophan
AA: Amino acids
ABCA1: ATP-binding cassette transporter A1
ALP: Alkaline phosphatase
ALT: Alanine transaminase
ApoE: Apolipoprotein E
AST: Aspartate transaminase
BA: Bile acid
BAS: Bile acid sequestrant
BBR: Berberine
B/B ratio: Bacillota/Bacteroidota ratio
BSH: Bile salt hydrolase
BW: Body weight
CXCL: Chemokine (C-X-C motif) ligand
CYP7A1: Cholesterol 7α-hydroxylase
EGCG: Epigallocatechin gallate
FAO: Hepatic fatty acid oxidation
FFA: Free fatty acids
FGF19: Fibroblast growth factor 19
FGFR4: Fibroblast growth factor receptor 4
FMO3: Flavin-containing monooxygenase 3
FXR–SHP: Farnesoid X receptor–small heterodimer partner
GABA: Gamma-aminobutyric acid
Gcg: Glucagon
GLP-1: Glucagon-like peptide 1
GT51: Glycosyltransferase family 51
HCD: High-cholesterol diet
HDL: High-density lipoprotein
HFD: High-fat diet
HMGCR: 3-hydroxy-3-methylglutaryl coenzyme A reductase
HMW: High molecular weight
HNF4α: Hepatocyte nuclear factor 4α
IgA: Immunoglobulin A
IL: Interleukin
LDL: Low-density lipoprotein
LDLR: Low-density lipoprotein receptor
LPS: Lipopolysaccharide
LPC: Lysophosphatidylcholine
Mdr: Multidrug-resistant
NAFLD: Non-alcoholic fatty liver disease
NEFA: Non-esterified fatty acids
NF-κB: Nuclear factor kappa B
NPC1L1: Niemann–Pick C1-like 1 protein
oxLDL: Oxidized low-density lipoprotein
PCSK9: Proprotein convertase subtilisin/kexin type 9
PGC: PPARγ coactivator
PPAR: Peroxisome proliferator-activated receptor
PS: Phytosterols
RCT: Reverse cholesterol transport
RYR: Red yeast rice
SCFA: Short-chain fatty acids
SD: Sprague–Dawley
SHIME: Simulator of the human intestinal microbial ecosystem
SIRT1: Sirtuin 1
SREBP: Sterol regulatory element-binding protein
TC: Total cholesterol
TCA: Tauro-cholic acid
TG: Triglycerides
TJ: Tight junction
TLR: Toll-like receptor
TMA: Trimethylamine
TMAO: Trimethylamine N-oxide
TNF: Tumor necrosis factor
UFA: Unsaturated fatty acids
VLDL: Very low-density lipoprotein

## Declarations

The figures in this article were created using online graphic design tool Canva (Canva Pty Ltd., Australia).

The authors declare that no generative AI or AI-assisted technologies were used in the preparation of the manuscript.
